# Bacterial hazard identification and exposure assessment of raw milk consumption in Jimma zone, South West Ethiopia

**DOI:** 10.1186/s12866-023-02910-0

**Published:** 2023-06-13

**Authors:** Beje Gume, Leykun Berhanu, Tesfaye Kassa, Habib Bediru, Assegid Getaneh Fikre, Lelisa Sena Dadi, Seid Tiku Mereta

**Affiliations:** 1 Department of Environmental Health Science and Technology, Jimma University, P.O. Box 378, Jimma, Ethiopia; 2 Department of Environmental Health Science, Wollo University, P.O. Box 1145, Dessie, Ethiopia; 3 School of Medical Laboratory Science, Jimma University, P.O. Box 788, Jimma, Ethiopia; 4 Jimma Town Livestock and Fishery Development Office, P.O. Box 378, Jimma, Ethiopia; 5 School of Veterinary Medicine, Jimma University, P.O. Box 307, Jimma, Ethiopia; 6 Department of Epidemiology, Jimma University, P.O. Box 378, Jimma, Ethiopia

**Keywords:** Dairy farm, Microbial hazard, Distributers, Retailer outlet

## Abstract

**Background:**

Raw milk may contain pathogenic microorganism that can sometimes fatally affect the health of consumers. However, risks related to raw milk consumption in Southwest Ethiopia are not well studied. The aim of this study was to evaluate the presence of five target pathogenic bacteria including *Escherichia coli* O157:H7, *Salmonella* enterica *Typhimurium*, *Staphylococcus aureus*, *Listeria monocytogenes*, and *Campylobacter jejuni* in raw milk and to assess exposure associated with the consumption of raw milk.

**Method:**

A cross-sectional study was carried out between November 2019 and June 2020 to in Jimma zone, Southwest Ethiopia. Laboratory analysis was conducted on milk samples collected from Seven Woreda towns, including, Agaro, Yebu, Sekoru, Serbo, Shebe, Seka, Sheki and Jimma town administration. Semi-structured interview questions were administered to collect data on the amount and frequency of consumption. Descriptive statistics were used to summarize laboratory results and questionnaire survey data.

**Result:**

Among 150 total raw milk samples, about 61.3% were found contaminated by one or more types of pathogens along the dairy value chain. The highest and the least bacterial counts recorded were 4.88 log_10_cfu/ml and 3.45 log_10_cfu/ml from *E. coli* and *L. monocytogenes* respectively. The mean concentrations of pathogens demonstrated significant statistical difference (*p* < 0.05) using 95% confidence interval where the prevalence percentage of isolates increased as the milk was transported from farms to the retail outlets. Except for *C. jejuni*; all other pathogens were detected in the range of unsatisfactory level of milk microbiological quality along the chain. The estimated mean annual risk of acquiring intoxication of *E. coli* across retailer outlets is 100% whereas salmonellosis, *S. aureus* intoxication, and listeriosis are 84%, 65% and 63% respectively.

**Conclusion:**

The study highlights the significant health risks associated with the consumption of raw milk due to its unacceptable microbiological quality. The traditional production and consumption patterns of raw milk are the primary reasons for the high annual probability of infection. Therefore, regular monitoring and implementation of hazard identification and critical control point principles are necessary from raw milk production to retail points to ensure the safety of consumers.

## Introduction

The increasing magnitude of microbial contamination of food is among the most important challenges associated to public health, since the hazard existence has been recognized [34]. Animal products are generally considered as high risk food items with respect to pathogen contents, natural toxins and other unavoidable pre-processing and post-processing activities [12]. Milk and milk-based food products are highly susceptible to microbial contamination because of their rich composition, which provides a favorable medium for growth of spoilage agents [4].

The most predominant zoonotic bacterial contaminants of raw milk and other dairy products include *Staphylococcus aureus*, *Escherichia coli*, *Salmonella* enterica *typhimurium*, *Shigella spp.*, *Listeria monocytogenes*, *Yersinia enterocolitica*, *Campylobacter jejuni*, *Streptococcus spp.*, and *Pseudomonas spp.* [27, 37, 38]. Foods contaminated with such biological hazards and ready for consumption are one of the health concerns in the world causing food-born health problems. Ethiopia shares the same global health concerns associated to raw milk consumption, mainly gastroenteritis and typhoid fever as the rest of the world. Food-borne gastrointestinal disease can be either due to infection by vegetative bacterial cells or by intoxication from toxigenic bacteria such as *S. aureus* and *E. coli*. Food-borne illnesses create an enormous burden on the country’s economy such as consumer costs including medical, legal, and other expenses, as well as absenteeism at work and school [2]. In relation to food-borne illness, World Health Organization (WHO) and the Food and Agriculture Organization (FAO) have set standards for acceptable daily intake of microbial load in foods in order to safeguard human health [12].

Even though raw milk is considered as relatively free from contaminants when it leaves the udder, it is highly prone to the invasion of various exogenous pathogenic bacteria [22, 26, 27, 36, 37]. It has been reported between 2006 and 2012 that twenty outbreaks of illness have been associated with the consumption of raw milk in New Zealand [27].

Milk is an important source of nutrition in developing countries like Ethiopia. Although its milk yield is found to be low, Ethiopia ranks first in the number of livestock in Africa [25]. It is estimated that Jimma zone has 456,893 heads of cattle, which is relatively the largest cattle population in Oromia region [3]. Hence, the people are highly dependent on the foods of animal source, mainly on dairy products. Despite the preference of consuming raw milk and other dairy products in the community, there is no formal standard followed to maintain the hygienic conditions of milk along the production and transportation line. Hand milking, unsafe transportation under ambient temperatures and collection of milk in unsterile containers are the common practices among the community that lead to poor milk hygiene. Microorganisms take advantages of time for proliferation all the way through such unhygienic production, transportation and storage [6, 13].

In spite of these unhygienic practices that could pose potential health problems in our community, there are very few related studies assessing biological hazards and public exposures. In addition, Ethiopia has no systematic surveillance and management systems that help in identifying, quantifying and controlling food safety hazards and potential adverse health effects resulting from human exposure [2]. It is necessary,however, to quantify biological hazards in readily consumable foods and to have baseline information on the risk factors related to food contamination. Therefore, the objective of this study is to identify and characterize hazards and to assess exposure associated with the consumption of raw milk in Jimma zone.

## Methods

### The study area, sampling and design

A cross sectional study was carried out in Jimma zone, Southwest Ethiopia located about 350 km from Addis Ababa, the capital of the country. The elevation of Jimma Zone varies from 1000 to 3360 m above sea level. According to the 2007 national population and housing census, the zone has a total population of 2.6 million, of which 88.7% are rural residents. A total of 521,506 households were counted in this zone, which results in an average of 4.77 persons to a household, and 500,374 housing units. Jimma zone has estimates of 456,893 heads of cattle [5, 7]. It is estimated that about 203L of milk is produced in Jimma Zone per one lactation period of a cow [19]. Seven Woreda (a geo-political sub division of a zone) towns including Agaro, Yebu, Sekoru, Serbo, Shebe, Seka, Sheki and Jimma town administration were purposely selected for this study.

### Sample collection

**Fig. 1:**
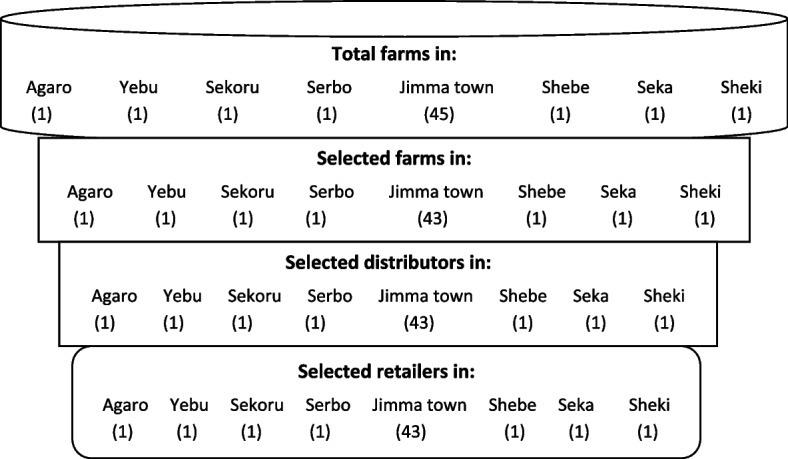
Simplified diagrrram showing milk collection points; farms, distribution centers, and retailer outlets and sample size

A total of 150 milk samples were collected from sampling points; 50 from dairy farms (a sample from each Woreda center and 43—samples from Jimma town administration), 50 from collection and distribution centers of Jimma town administration, and 50 from retail outlets of selected Woreda towns and Jimma town administration (a sample from each Woreda center and 43—samples from Jimma town administration). The samples were collected from all small to large scale urban dairy farms, examining the value chain from farm to table; nearly all farms within the selected study area were included in the analysis (Fig. 1).

About 25 ml of raw milk samples were collected aseptically with sterile universal screw capped plastic bottles using sterilized 50 ml syringe. The samples were collected at dairy farms immediately after milking from the storage containers ready to be transported. At distribution centers and retailer outlets, the samples were taken directly from milk storage tanks immediately after the milk had been transported and collected in the tanks. At each stage of dairy supply chain, a sample was taken from two to three randomly selected containers within the same sterile bottle. All collected samples were labeled, placed in cold ice boxes and transported within 4 h of sampling to the Microbiology Laboratory School of Medical Laboratory Sciences in Jimma University for microbiological analysis.

### Questionnaire and interview survey

As there is no reliable estimate on the exposure status of inhabitants of Jimma zone for microbial contamination through raw milk consumption, a 50% proportion which leads to the highest possible sample size was used as recommended by Daniel [8]. The estimate is desired to be with 5% margin of error and 95% confidence interval. Therefore the required sample size for this study was determined using the following statistical formula:

$$\mathrm{n}=\frac{{\mathrm{NZ}}^{2}\mathrm{pq}}{{\mathrm{d}}^{2}\left(\mathrm{N}-1\right)+({\mathrm{Z}}^{2}\mathrm{pq})}$$

where “N” is the total number of households in towns of Jimma zone which is 60,107, “n” is minimum number of sample size, “Z” is standard normal score, “p” is the prevalence value, “q” = 1-p and “d” is marginal error. At 95% confidence interval Z = 1.96 and marginal error is 5%. Since no report is yet recorded for prevalence of exposure the *p-value* is considered to be 50%. Considering 10% non-response rate, the final sample size for raw milk exposure assessment is calculated to be 420. Interview questions were administered to collect data on the amount and frequency of consumption of raw milk so as to determine the exposure status. Trained data collectors who can understand both English and the local languages of the study area (mainly Oromifa) interviewed volunteer respondents. A total of four hundred and twenty milk consumers were interviewed on the spot of consumption from randomly selected 50 milk retail outlets (restaurants, cafeterias, hotels) along the chain of dairy clients.

### Microbial isolation and quantification

The bacterial load estimation per 1 ml of milk was done by mixing 25 ml of milk samples into 225 ml of sterilized buffered peptone water and were thoroughly shaken to make one-in-ten initial dilution of the sample; the stock solution. Ten-folds of serial dilutions were made from the homogenates up to 10^–6^ with three replicates each except for *Salmonella*. Appropriate spread plates were made with 0.1 ml aliquots from all serial dilution tubes and incubated at appropriate temperature for the pathogen of an interest. Bacterial colonies were counted using colony counter to determine colony forming units (cfu)/ml. Dilutions with the total number of colonies on a plate were used for cfu computation according to the following formula.

$${}^{CFU}\!\left/ \!{}_{mL}\right.=\frac{\mathrm{ No\ of\ Colony\ counted\ on\ plates }}{\mathrm{volume\ plated}\left(\mathrm{mL}\right)*\mathrm{dilution\ factor}}$$

*Salmonella* spp. were detected from milk samples by the conventional microbiological analysis according to International Standards Organization ISO-6579–2002 methods using buffered peptone water as pre-enrichment medium. Selenite Fraser Broth (SFB) was used as both selective enrichment and enumeration medium of salmonella with Most Probable Number (MPN) tubes. The probable numbers of colony forming units of salmonella was determined by turbidity of the MPN tubes. For isolation of *Salmonella*, Salmonella-Shigella agar (SSA) was used as selective plating. One milliliter of a stock solution was transferred into SFB and incubated for 24 h followed by which a loop full of SFB was streaked on SSA and incubated for 24 h at 37 °C. Finally, black colonies on SSA were identified as presumptive colonies of *Salmonella*. Finally, Indole Methyl-red Voges-Proskauer Citrate IMVC test was applied for biochemical confirmation of salmonella. *Staphylococcus* spp. were isolated and enumerated by spread plate on Mannitol-salt agar (MSA) using American Public Health Association-2001 method. After 24 h incubation of spread plates at 37 °C, golden yellow colonies were considered as presumptive *S. aureus.* Grams staining and coagulase tests using plasma [28, 31] were used as confirmation of the presumptive colonies**.** Gram positive and coagulase-positive colonies were considered as *S. aureus*. Buffered peptone water was used as primary enrichment followed by Buffered Listeria Enrichment Broth (BLEB) as secondary enrichment to isolate *Listeria* spp. The preparations were incubated at 30 ^0^C for 48 h. Listeria selective agar base (LSA) enriched with sheep blood and Listeria selective supplements were used as a selective differential medium for *L. monocytogenes*. The spread plate cultures were incubated for 37 ^0^C for 48 h and β-hemolytic colonies on LSA were identified as *L. monocytogenes*. Gram staining, microscopy and catalase tests were performed for morphological characterization and confirmation. Isolation and enumeration of presumptive *E. coli* in meat sample was done by plating dilutions on MacConkey Sorbitol Agar (MacSA, Oxoid, UK). The characteristic colonies that are identified as presumptive *E. coli* O157:H7 were sub cultured on Eosin Methylene Blue Agar (EMBA, Oxoid, UK) and aerobically incubated at 37 °C for 24 h after incubation which the colonies with distinct metallic sheen were counted. IMViC test was also applied for biochemical confirmation. Campylobacter Selective Agar base (CSA) enriched with sheep blood and Campylobacter selective supplement was used as a selective differential medium for isolation of *Campylobacter* spp. Because of cellular sensitivity outside the gastrointestinal environment resulting in possible sub-lethal effects on the cells, a loop full milk sample were streaked directly from the stock solution onto enriched CSA. The cultures were incubated in moist anaerobic jar at 40^0^C. Grayish flat colonies growing on the medium were considered as presumptive *C. jejuni*. Further morphological (microscopic) examination and biochemical tests (Gram’s, catalase, H_2_S and oxidase tests) were done to confirm *C. jejuni*.

### Microbial hazards and exposure assessment

The exponential model $\mathrm{P}\left(\mathrm{d}\right)=1-{\mathrm{e}}^{-\mathrm{rd}}$ [33] was used to estimate the probability of infection through consumption of contaminated milk,where, “P(d)” is probability of infection at dose (d) per serving, “d” is the mean dose (cfu) of pathogens consumed per person per a day (microbial infection, “r” is dimensionless infectivity constant. The model parameter “r” was 2.18 × 10^–4^ for *E. coli*, 3.97 × 10^–6^ for *Salmonella*, 7.64 × 10^–8^ for *Staphylococcus*, 3.29 × 10^–7^ for *Listeria*, and 2.44 × 10^–8^ for *Campylobacter* [9, 10, 12, 14, 32]. The duration, number of times of raw milk consumption practice (frequency) and the amount of milk consumed in a given period (quantity) were identified and the information was combined to estimate the level of exposure of study subjects to the microbiological hazards. The annual probability of infection was calculated from the probability of infection and the number of days of exposure within the year according to the relation: P_(ann)_ = 1-(1-P(d))^n^ [33],where “n” is the number of days of exposure within the year.

### Quality assurance

Sample collection and laboratory analyses were carried out under close supervision. All media and reagents used were up to date, and all microbiological analysis was carried out inside level II biosafety cabinet (BDK, Genkingen, Germany). All culture media and materials were sterilized using autoclave (Astell, England). The adequacy of sterilization was also assured using sterilization indicator.

### Data management and statistical analysis

Laboratory results and questionnaire survey data were stored in Microsoft Excel spreadsheet. The statistical data analysis was done by using Statistical Package for Social Science (SPSS) version 23. Descriptive measures including mean, percentage and frequency were used to quantify the level of contamination of milk by the photogenic bacteria. The microbial load measurement was normalized to cfu/ml converting into log 10 values. Statistical differences among the mean concentrations of pathogens along the dairy value chain were assessed using *p-values* by which values less than 0.05 were considered as significant.

## Results

### Hazard identification and milk microbial quality

#### Frequency of milk contamination along the dairy value chain

**Fig. 2:**
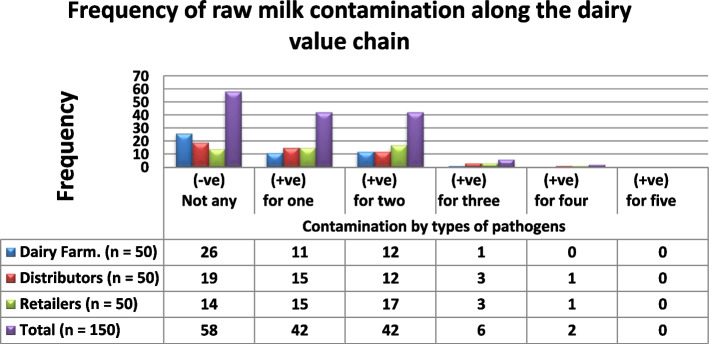
Frequency of microbial contamination of mil along the dairy value chain; (-ve) = negative; (+ ve) = positive

Among the total of 150 raw milk samples collected, 92 (≈61.3%) were found contaminated along the dairy value chain. One or more types of pathogens were found to be in a milk sample analyzed in the laboratory. A total of 42 (28%) samples contained at least one type of pathogens, whereas 2(1.3%) samples contained four different types of pathogens of interest in this study (Fig. 2).

#### Incidence of specific pathogens in milk samples along the value chain

Out of the total of 150 raw milk samples analyzed in the laboratory, 54 (36%) were positive for *E. coli*; 30 (20%), 58 (38.7%), 8 (5.3%), and 2 (1.3%) were positive for *Salmonella*, *S. aureus*, *Listeria*, and *Campylobacter* isolates respectively. In all cases, the microbial contamination incidence (percentage) of the samples appeared to increase from dairy farms to the retail outlets except for *Campylobacter* isolates (Table 1).

**Table 1:** Incidence (%) of milk contamination by pathogens along the dairy value chain

**Pathogens**	**Farms** ***n* = 50**	**Distributors** ***n* = 50**	**Retailers** ***n* = 50**	**Total** ***n* = 150**
	** + ve (%)**	** + ve (%)**	** + ve (%)**	** + ve (%)**
*E. coli*	14(28%)	18(36%)	22 (44%)	54 (36%)
*Salmonella*	7 (14%)	11 (22%)	12 (24%)	30 (20%)
*S. aureus*	16 (32%)	18 (36%)	24 (48%)	58 (38.7%)
*L. monocytogenes*	1 (2%)	3 (6%)	4 (8%)	8 (5.3%)
*Campylobacter*	0 (0%)	2 (4%)	0 (0%)	2 (1.3%)

** + ve**: *positive samples*

The logarithmic means of pathogenic bacteria counts in all milk samples ranged from 1.48 log_10_cfu/g (30 cfu/ml) to 4.88 log_10_cfu/ml (7.50 × 10^5^ cfu/ml). The highest bacterial count was recorded from *E. coli* contamination ranging from 2.64 log_10_cfu/ml (4.36 × 10^3^ cfu/ml)—4.88 log_10_cfu/ml (7.50 × 10^5^ cfu/ml) whereas the lowest from *Campylobacter* which was 1.48 log_10_cfu/ml (30 cfu/ml) found only at distributer points. The counts of *Salmonlla*, *S. aureus*, and *L. monocytogenes* ranged from 2.53 log_10_cfu/ml (3.40 × 10^3^ cfu/ml)—4.04 log_10_cfu/ml (1.10 × 10^5^ cfu/ml), 2.89 log_10_cfu/ml (7.68 × 10^3^ cfu/ml)—4.65 log_10_cfu/ml (4.43 × 10^5^ cfu/ml), and 2.32 log_10_cfu/ml (2.10 × 10^3^ cfu/ml)—3.45 log_10_cfu/ml (2.80 × 10^4^ cfu/ml) respectively. Samples from retail outlets showed the highest bacterial counts, where as those from farms where the least; except for *Campylobacter* counts, in which the case appeared only at distributer points (Table 2).

**Table 2:** Mean concentration counts of pathogenic bacteria along the dairy value chain

**Pathogens**	**Dairy farms**		**Distributers**		**Retailers**	
	**cfu/ml**	**Log**_**10**_**cfu/ml**	**cfu/ml**	**Log**_**10**_**cfu/ml**	**cfu/ml**	**Log**_**10**_**cfu/ml**
*E. coli*	4.36 × 10^3^	2.64	5.82 × 10^4^	3.76	7.50 × 10^5^	4.88
*Salmonella*	3.40 × 10^3^	2.53	5.16 × 10^4^	3.71	1.10 × 10^5^	4.04
*S. aureus*	7.68 × 10^3^	2.89	4.89 × 10^4^	3.69	4.43 × 10^5^	4.65
*L. monocytogenes*	2.10 × 10^3^	2.32	9.67 × 10^3^	2.99	2.80 × 10^4^	3.45
*Campylobacter*	–-	–-	30	1.48	–-	–-

#### Microbial quality of milk samples along the dairy value chain

The pathogens were detected in the range which unfit for human consumption (unsatisfactory level) at each point along the dairy value chain except for *C. jejuni*; which were isolated only from distributors (Table 3). However, the incidence of pathogens was varied that 48% of samples were found contaminated with *S. aureus* in the range of unsatisfactory level among consumable samples at retailers. But none of the samples was recorded from *C. jejuni* contamination*. E. coli*, *Salmonella* and *L. monocytogenes* were 44%, 24%, and 8% respectively.

**Table 3:** Microbiological quality of milk samples along the DVC according to the guideline interpretation of results of specific food-borne pathogens in RTE foods (CFS, 2014)

**Microbiological quality guideline category**	**Guideline limits**	**% microbiological quality along the DVC**		
		**Farms**	**Distributers**	**Retailers**
	**cfu ml**^**−1**^	***n* = 50**	***n* = 50**	***n* = 50**
*E. coli*				
SA	< 20	72	64	56
BL	20—≤ 10^2^	0	0	0
US	> 10^2^	28	36	44
*Salmonella*				
SA	n.d. in 25 ml	86	78	76
BL	N/A	-	-	-
US	d. in 25 ml	14	22	24
*S. aureus*				
SA	< 20	68	64	52
BL	20—≤ 10^4^	18	6	0
US	> 10^4^	14	30	48
*L. monocytogenes*				
SA	< 10	98	94	92
BL	10—≤ 10^2^	0	0	0
US	> 10^2^	2	6	8
*Campylobacter*				
SA	n.d. in 25 ml	100	96	100
BL	N/A	-	-	-
US	d. in 25 ml	0	4	0

*RTE* Ready-to Eat, *DVC* Dairy value chain, *SA* Satisfactory, *BL* Boarder line, results indicating the upper limit of acceptability and potential development of unacceptable risk, *US* Unsatisfactory, results indicating potentially injurious level to health and/or unfit for human consumption, *N/A* Not applicable

### Dose–response and exposure assessment

#### Raw milk consumption and exposure practice

A socio-demographic report of 420 raw milk consumers revealed that 254 were male and 166 were female. In terms of age, 6 were less than or equal to 18, 353 were greater than up to less than or equal to thirty, and 61 were greater than 30. Educational status showed that 13 were illiterate, 29 were primary, 71 were secondary, and 307 were tertiary. Religion-wise, 235 were Christian, 176 were Muslim, and the rest 9 were others. Over two-third (75.7%) of respondents had the custom of drinking raw milk. Majority (45.7%) of them experienced long period raw milk consumption, at least once in a week, and chiefly 300 ml per serving (69.5%). It was reported that most respondents prefer raw milk, mainly in the form of homemade yoghurt, for its taste (69.5%), minority (2.9%) for it is tradition of their community, and some others for both reasons (3.3%). Contrastingly, respondents also reported their experience of disease symptoms due to consumption of raw milk (Table 4).

**Table 4:** Raw milk consumption details of respondents in Jimma, Southwest Ethiopia

**Parameter categories**	**Frequencies (*n* = 420)**	**Percentages (*n* = 420)**
Raw milk consumption experience		
Yes	318	75.7
No	102	24.3
Type of raw milk consumed		
Never	102	24.3
Whole milk	18	4.3
Homemade yoghurt	300	71.4
Duration of raw milk consumption experience (in years)		
Never	102	24.3
< 1 year	21	5.0
1–5 years	43	10.2
5–10 years	62	14.8
> 10 years	192	45.7
Frequency of raw milk consumption (days/week)		
Never	102	24.3
1 day	171	40.7
2 days	136	32.4
3 days	7	1.6
4–7 days	4	1.0
Amount of raw milk consumed per serving (in L)		
Never	102	24.3
0.30	292	69.5
0.50	26	6.2
Reason for raw milk consumption		
Never	102	24.3
Culture	12	2.9
Taste	292	69.5
Both culture & taste	14	3.3
Knowledge and/or experience of disease due to raw milk consumption		
Never	102	24.3
Abdominal discomfort	103	24.5
Nausea	112	26.7
Vomiting	86	20.5
Diarrhea	17	4.0
**Average total**	**420**	**100**

#### Dose response to pathogen exposure

Estimates of most of the pathogens in the serving units of raw milk were too large that the highest (7.50 × 10^5^ cfu) recorded from *E. coli* and the least (2.80 × 10^4^ cfu) from *L monocytogenes*. Probability of infection at the consumption dose shows very high that the annual probability of infection is perfectly-1 in the cases of four pathogens. But, none of samples from retail outlets contained *C. jejuni* (Table 5).

**Table 5:** Exposure dose and risk of infection due to consumption of raw milk

Pathogens	*d*	*P(d)*	*P*_*(ann)*_
	***Cfu/ml***	**1-e**^**−rd**^	**1-(1-p(d))**^**n**^
*E. coli*	7.50 × 10^5^	1	1
*Salmonella*	1.10 × 10^5^	0.84	1
*S. aureus*	4.43 × 10^5^	0.65	1
*L. monocytogenes*	2.80 × 10^4^	0.63	1
*Campylobacter*	0	0	0

d = mean dose of pathogens (cfu) consumed per serving unit; P(d) = probability of infection at dose d; P(ann) = annual probability of infection due to exposure

## Discussion

Results of this study revealed the contamination of raw milk by either one or a combination of pathogenic bacteria. The number of contaminated raw milk samples was found to be significantly high (61.3%). This figure is virtually comparable to the 62.5% contamination in the distribution center milk containers in Mekelle, Ethiopia [11] and 65.5% of contamination reported from parallel study conducted in selected street foods in Gondar Town [1]. The chance of contact of raw milk to different types of bacterial pathogens increased from farms to the retail outlets along the dairy value chain. This can be associated to the advantage that microorganisms take, having different contact surfaces and time through the milk production chain, from farm-to-table.

Except for the isolates of *C. jejuni*, which appeared only in the samples from distributers, all other pathogenic bacteria were identified in the milk samples from every source; dairy farms, distributers and retail outlets. The load of *E. coli* was ranged from 2.64–4.88 log_10_ cfu/ml, where the mean concentration was 3.76 ± 1.12 log_10_ cfu/ml (mean ± SD). This is the highest incidence recorded in our study as shown in Table 2. The result from *E. coli* contamination in this study is comparable to 2.00–6.07 log_10_ cfug^−1^ reported from the rural household served foods in Malawi by Taulo et al., [35]. Sabuj et al., [32] also reported 4.10–4.58 log_10_ cfug^−1^ *E. coli* from street-vended ready-to-eat foods in Bangladesh Agricultural University. Next to the eminent raw milk contaminant pathogens considered in this study, *E. coli*, were *S. aureus*, *S.* enterica *Typhimurium*, *L. monocytogenes*, *C. jejuni* that appeared to be with mean concentrations of 3.74 ± 0.88 log_10_ cfu/ml, 3.42 ± 0.79 log_10_ cfu/ml, 2.92 ± 0.56 log_10_ cfu/ml, and 1.48 log_10_ cfu/ml respectively. The total absence of *C. jejuni* from the retail outlets in our study directly coincides with its zero occurrences reported by Taulo et al. [35] in ready-to-eat foods,which can be ascribed to the fastidious nature of the organism.

*S. aureus* was the second prevalent pathogen determined in our study followed by *E. coli*. The mean concentration of *S. aureus* surpassed 10^5^ cfu/ml in the samples from retailers, which could have posed potential risk among raw milk consumers. Studies conducted by Heidinger et al., [17] and Giacometti et al., [15] in raw milk also revealed parallel prevalence of *S. aureus. S.* enterica *Typhimurium* and *L. monocytogenes* had relatively lower mean concentration, each having 3.42 ± 0.79 log_10_ cfu/ml and 2.92 ± 0.56 log_10_ cfu/ml respectively. Studies also revealed corresponding results for high *Salmonella* contamination [16, 29]. However, the figures of prevalence of *L. monocytogenes in* this study are considerably large in relation to its very low (0.08%) to zero occurrences [21, 24].

Generally, the prevalence and percentages of most isolates of pathogens considered in our study increased as milk transported from farms to the retail outlets (Table 1). This could be connected with the contamination of bacteria through milking, collection, and transportation processes and the proliferation of bacteria using the opportunity of time from production and processing to the table for consumption. Except for *C. jejuni*, the mean concentrations of all pathogens demonstrated significant statistical difference (*p* < 0.05) using 95% confidence interval along the dairy value chain.

Except for the *C. jejuni*, all pathogens considered in this study were detected in the unsatisfactory level along the dairy value chain, particularly at retail outlets, where raw milk is ready-to-eat. It is alike the result in the study conducted by Manguiat and Fang [24] on street vended foods where most of the pathogens found in unsatisfactory levels. The laboratory analysis of samples (48%) showed that *S. aureus* were present in the ranges of 10^5^ cfu/ml,which is certainly large number compared to the unsatisfactory level (> 10^4^ cfu/ml) rated in guide lines [6]. Odu and Assor [30] also obtained corresponding results (42.1%) from *S. aureus* contamination. *E. coli* were detected in 44% of milk samples from retailers, against the limits of guidelines, in which their presence in > 10^2^ cfu/ml considered as potentially hazardous. *S.* enterica *Typhimurium* (24%) and *L. monocytogenes* (8%) were present in the ranges of 10^5^ cfu/ml and 10^4^ cfu/ml respectively. According to the standard limits of guidelines [6], however, the ranges determined for both pathogens is classified as unsatisfactory.

In this present study, the survey of raw milk consumption data on the amount, frequency, and duration was translated for the estimates of the number of pathogens present in the milk. These variables were significantly associates with the annual probability of infection (Table 5) among the study population. An exponential dose–response model was used, assuming quantitative estimates of pathogens at the contaminated milk portion size (serving units) ingested by the presumptive population at risk [20, 32, 33]. The mean amount of raw milk consumed (0.31 ± 0.05 L) had evidently direct relation to the large number of pathogens in the serving units in addition to the chance of proliferation that the pathogens could have along the dairy chain to the retailer outlets. In this study, therefore, the probable dose of pathogens assumed was the product of cfu/ml in raw milk and the mean amount of milk consumed at once. The results of the current study, accordingly, demonstrated ingestion of very high number of pathogens with serving units of raw milk,2.37 × 10^8^ *E. coli*, 1.41 × 10^8^ *S. aureus*, 3.49 × 10^7^ *S.* enterica* T*., and 8.89 × 10^6^ *L. monocytogenes*, (Table 5). Similarly, [32], Manguiat and Fang 2013, [18, 29] reported high ranges of pathogen contamination in their findings. In general, our study reflected that the microbiological quality of raw milk in the study area was considerably unsatisfactory with respect to pathogens treated. Mamun et al., [23] also reported high levels of potential hazards to the public health threats especially due to high number of coliforms,like *S. aureus*, *E. coli*, and *S.* enterica* T* in foods vended by school-based streets.

Except for *C. jejuni*, the annual probability of infection by all other pathogens is perfectly one; i.e. 100% (Table 5). This finding coincides with the 100% chance of being infected by potential pathogens as reported in previous studies [39] and [32]. Raw milk consumers had experience and/or information of disease symptoms like nausea (26.7%), abdominal discomfort (24.5%), vomiting (20.5%) and diarrhea (4.0%). This could be associated to either one or combination of intoxications of *E. coli* and *S. aureus*, salmonellosis, and listeriosis. The probability of infection at consumption dose was also high regardless of the category of consumers varied in experience of duration, amount and reason for raw milk consumption. Generally, the estimated risk at consumption dose of acquiring intoxications of *E. coli* across retailer outlets is 100%, whereas salmonellosis, *S. aureus* intoxication and listeriosis is 84%, 65% and 63% respectively.

## Conclusion

Our study specifically found that raw milk in the dairy value chain in Jimma, Southwest Ethiopia, is contaminated with almost all types of bacterial hazards. The prevalence of frequent bacterial contaminants and the deterioration of raw milk across retail outlets were also demonstrated. Over half of the total samples had high level of pathogenic bacteria, most of which were found to be in the range of unsatisfactory limits that could pose health risks to consumers. Therefore, regular monitoring and implementing hazard identification and critical control point principles along the dairy value chain are necessary to ensure the safety of raw milk. These findings highlight the need for immediate action to prevent the spread of foodborne diseases caused by bacterial hazards in food.

## Data Availability

All data and materials are available for this work and can be accessed from the corresponding author.
